# Reducing the Meta-Emotional Problem Decreases Physiological Fear Response during Exposure in Phobics

**DOI:** 10.3389/fpsyg.2016.01105

**Published:** 2016-07-25

**Authors:** Alessandro Couyoumdjian, Cristina Ottaviani, Nicola Petrocchi, Roberta Trincas, Katia Tenore, Carlo Buonanno, Francesco Mancini

**Affiliations:** ^1^ENPlab, Department of Psychology, Sapienza University of RomeRome, Italy; ^2^Neuroimaging Laboratory, IRCCS Santa Lucia FoundationRome, Italy; ^3^Scuola di Psicoterapia Cognitiva S.r.l.Rome, Italy

**Keywords:** specific phobia, meta-emotional problem, double standard, phobic stimuli, heart rate, heart rate variability, autonomic nervous system, self-criticism

## Abstract

Anxiety disorders may not only be characterized by specific symptomatology (e.g., tachycardia) in response to the fearful stimulus (primary problem or first-level emotion) but also by the tendency to negatively evaluate oneself for having those symptoms (secondary problem or negative meta-emotion). An exploratory study was conducted driven by the hypothesis that reducing the secondary or meta-emotional problem would also diminish the fear response to the phobic stimulus. Thirty-three phobic participants were exposed to the phobic target before and after undergoing a psychotherapeutic intervention addressed to reduce the meta-emotional problem or a control condition. The electrocardiogram was continuously recorded to derive heart rate (HR) and heart rate variability (HRV) and affect ratings were obtained. Addressing the meta-emotional problem had the effect of reducing the physiological but not the subjective symptoms of anxiety after phobic exposure. Preliminary findings support the role of the meta-emotional problem in the maintenance of response to the fearful stimulus (primary problem).

## Introduction

Emotions are complex and multidimensional: we not only feel an emotion, such as anger, fear, or sadness as a response to a stimulus, but we also judge and feel emotions about our emotions. Perceptions and appraisals of emotional states have long been recognized and described as meta-experiences of mood and emotions (Mayer and Gaschke, 1988) or meta-emotions (Mendonca, 2013). Perceiving an emotion as problematic, aversive, or unacceptable, instead of normal, comprehensible, and acceptable can influence the way a person regulates the emotional state itself (Gardner et al., 1988; Hofmann, 2013).

From a clinical point of view, it is commonly accepted that patients may “disturb themselves about their disturbances” (Dryden, 2000; p. 55). This phenomenon has been defined as *secondary problem* or *meta-emotional problem* (Ellis, 1980, 2003), and it is considered a common feature of many affective disorders. For example, a client might be depressed not only because he judges his layoff as a sign of being a loser, but also because he considers his depressive reaction (e.g., loss of interest and reduced energy) further evidence of personal failure. He unintentionally gives himself a ‘double dose’ or ‘two problems for the price of one’ (Dryden, 2000). As a common example, depressive rumination, a key risk factor for clinical depression, is related to negative thinking about depressive symptoms (Nolen-Hoeksema, 1991, 2000). Similarly, social phobics often worry about the negative consequences of their anxiety in social contexts (e.g., being judged as weakling or stupid; DSM-V; American Psychiatric Association [APA], 2013). The meta-emotional problem, other than being trans-diagnostic, seems one of the most relevant factors in psychopathology. For example, Clark and Beck (2010) claim that: “the greatest differences between clinical and non-clinical anxiety are evident in the secondary, strategic controlled processes responsible for the persistence of anxiety. For the clinical individual, further elaboration results in persistence and even escalation of anxiety, whereas the same processes result in reduction and possible termination of the anxiety program for the non-clinical person” (p. 53).

The role of negative self-evaluation in amplifying emotional reactivity has been indirectly confirmed by research on self-criticism. Self-criticism has mostly been described as a personality trait that leads people to experience frustration and anger toward themselves when they face setbacks and failures (Blatt, 2004; Gilbert et al., 2004). A growing number of studies confirmed that trait self-criticism is a *trans*-diagnostic phenomenon implicated in the development and maintenance of a range of psychological difficulties (Schanche, 2013) as it triggers, perpetuates, and intensifies emotional reactivity (Shahar, 2013). However, self-critical attitude has mostly been explored in relation to general life failures and setbacks, and research has mainly employed a self-report questionnaire. The key difference between self-criticism and the meta-emotional problem is that in the latter patients only criticize themselves for having a specific emotion, whereas self-criticism is much more pervasive and refers to all aspects of a patient’s life.

To the best of our knowledge, no studies have directly investigated how reducing negative evaluations (meta-emotional problem) of a specific negative emotional reaction (primary problem) impacts the experience of the emotional reaction by itself. In particular, there is no clear evidence that the decrease of the negative judgment of a phobic reaction has an impact on the primary emotional reaction that is the object of the critical judgment, especially in a clinical population. Whereas existing studies focused on the relationship between self-criticism and depressive symptomatology (e.g., Schanche, 2013), the relationship between the meta-emotional problem and anxiety symptomatology has been less explored. A noteworthy exception is provided by Wells’ Metacognitive Therapy (Wells, 2000) that has been applied in the context of both generalized anxiety and social phobia. The author found reductions in fear of negative evaluation after the administration of a brief metacognitive-focused treatment to such anxiety disorders (e.g., Wells, 2007). However, whereas the core target of Well’s Metacognitive Therapy is on erroneous beliefs about worry and unhelpful mental regulation strategies, the main goal of the present investigation is to investigate the meta-emotional problem in a broader sense and particularly its relationship to the primary problem. Additionally, while the metacognitive model focuses primarily on dysfunctional ways of relating to negative thoughts and beliefs, meta-emotional problems refer specifically to dysfunctional ways of evaluating and relating to negative emotional experiences. Moreover, a closer investigation of how a relatively simple and short intervention to change the evaluation of a phobic emotional reaction impacts the emotional reaction itself has not been conducted so far.

To investigate this process, we explored the effect of the decrease of the meta-emotional problem (e.g., ‘Having this fear is sign of weakness’) on anxiety symptoms (e.g., tachycardia) in patients with specific phobia (e.g., spider phobia). Specific phobia is one of the most prevalent mental disorders in the general population (Choy et al., 2007). This anxiety disorder is characterized by a marked and persistent fear of a specific object or situation (e.g., flying, heights, animals, receiving an injection, seeing blood), and generally patients recognize that their anxiety is excessive or unreasonable, and criticize themselves for being weak or for constantly overestimating how dangerous a situation is (DSM-V; American Psychiatric Association [APA], 2013).

Together with such subjective experience of heightened arousal, there is considerable evidence that subjects with specific phobia (animal sub-type) show increased heart rate (HR) and sympathetic activity during exposure to the phobic stimulus (e.g., Prigatano and Johnson, 1974; Sarlo et al., 2002). The sole focus on indices of sympathetic activity has, however, been highlighted as a limit of previous studies (Friedman and Thayer, 1998). During the past decades heart rate variability (HRV) has been increasingly used to understand the phenomenology of anxiety disorders, therefore in the present study we evaluated HR and measures of HRV as indices of autonomic nervous system responses to a phobic stimulus.

Our objective was to test if a brief intervention (Double Standard intervention; Leahy, 2003) designed to reduce negative self-evaluations in phobic patients may impact their reactivity to the phobic stimulus, both at subjective and psychophysiological levels. Double standard intervention is supposed to reduce negative self-evaluation by asking people to apply to themselves the same standards of evaluation that they would use to evaluate other people, which are usually milder (Leahy and Holland, 2000). We expected that, in comparison to a first exposure, a decrease in the meta-emotional problem would reduce the intensity of the emotional (levels of anxiety, disgust, etc.) and physiological (HR and HRV) fear reaction to the same phobic stimulus presented in a second exposure. This would provide preliminary evidence that modulating the negative evaluation of an emotional reaction impacts the experience of the emotional reaction itself, confirming the meta-emotional problem as one of the possible mechanisms responsible for the maintenance of phobic reactions.

## Materials and Methods

### Participants

Recruitment was conducted by flyers, websites, and social networks. The sample was composed of 33 participants, 6 men and 27 women, mean age 28.8 (9.8) years. The significant difference in gender reflects the fact that phobias are much more common in women than in men (e.g., Arnarson et al., 1998). All subjects were Caucasian and native Italian speaking. Exclusionary criteria were major psychiatric or cognitive problems requiring immediate treatment, psychotic or organic illnesses, substance abuse, cardiovascular disease, use of drugs/medications that may affect cardiovascular function, obesity (body mass index > 32 kg/m^2^), menopause, use of oral contraceptives during the previous 6 months, and pregnancy or childbirth within the last 12 months. People with flying phobia were excluded due to the practical difficulties with the exposure videos for these phobic stimuli. Injection phobia and blood phobia were excluded due to the peculiar autonomic nervous system reaction that characterizes these specific phobias (e.g., Sarlo et al., 2002; Ayala et al., 2010).

All participants had a Diagnostic and Statistical Manual of Mental Disorders (4th edition, DSM-IV; American Psychiatric Association [APA], 1994) diagnosis of Specific Phobia-Animal Type, as confirmed by administration of the Structured Clinical Interview for the DSM-IV (SCID-I; First et al., 1997) prior to data collection. The specific phobias distribution was as follows: spiders (*n* = 8), bugs (*n* = 6), bees (*n* = 4), cats (*n* = 3), grasshoppers (*n* = 2), dogs (*n* = 2), butterflies (*n* = 2), snakes (*n* = 2), pigeons (*n* = 2), insects (*n* = 1), lizards (*n* = 1). The Structured Clinical Interview for DSM-IV Axis II Personality Disorders Questionnaire and Interview (SCID-II; First et al., 1997) were also administered to assess potential co-morbidities. All patients were medication-free at the time of the study. The protocol was approved by the local Ethics Committee. Participants received 20 Euros as a compensation for their time.

### Video-Clips

**FIGURE 1:**
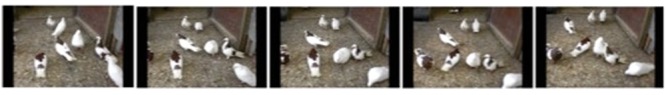
**Example of screenshot for the video-clip related to pigeon phobia**.

Each subject was presented twice with the same 30-s video-clip. Following the methodology used by Schaefer et al. (2014), each video-clip was selected from a variety of nature programs based on the specific phobia of the participant. Eleven exemplars of videos were selected including moving spiders, bugs, bees, cats, grasshoppers, dogs, butterflies, snakes, pigeons, insects, and lizards (see **Figure 1** for an example of the screenshot). For research purpose, the videoclips are available from the authors upon request. The video-clips used in the present study have not been previously validated; however, each of them has been preliminarily administered to a sample of 10 phobics and 10 non-phobic individuals eliciting significant differences in self-reported levels of anxiety and fear (*p*s < 0.0001).

### Questionnaires

After a phone pre-screening interview, a series of socio-demographic questions and the following questionnaires were administered on line via a survey system which guarantees the privacy and confidentiality of the respondents^1^:

(1) The Disgust Scale Revised (DS-R, Olatunji et al., 2007), which confirmed the Three-Factor Structure and showed good psychometric properties in its Italian translation (Olatunji et al., 2009);
(2) The Italian adaptation of the STAI (Spielberger et al., 1970) by Pedrabissi and Santinello (1989);
(3) The SCID-II Questionnaire (SCID-II-Q; First et al., 1997) administered on line with the aim to guide and shorten the subsequent interview.

Finally, participants were asked: “How much do you consider objectively dangerous the target of your phobia?”

Cronbach’s alpha coefficients for the DS-R, the STAI, and the SPQ were greater than 0.75 in the present study.

### Visual Analog Scales

During the experimental session, participants were asked to rate their current levels of feeling Happy, Sad, Anxious, Embarrassed, Angry, Calm, Dirty, Disgusted, and Ashamed on separate visual analog 100-point scales. The use of Visual Analog Scales to assess mood has been previously validated as a measure of emotional self-report in the context of anxiety (Rossi and Pourtois, 2012; Abend et al., 2014) and depression (van Rijsbergen et al., 2012, 2014). Given that disgust may play an important role in the physiological response to the phobic stimulus, levels of feeling Dirty were measured together with the commonly assessed mood. Moreover, given our specific interest in the meta-experience of mood and emotions, levels of being Embarrassed and Ashamed were also assessed.

After the first and second video presentations, participants were also asked to rate how much they considered objectively dangerous the phobic stimulus that they had just seen.

### Psychophysiological Measures

The electrocardiogram (ECG) was continuously monitored (Monitoring, Adatec s.r.l., Italy) with a standard electrode configuration (right clavicle and precordial site V6). Three disposable Ag/AgCl electrodes were used. Successive interbeat intervals (in milliseconds) within each period were written to single text files. Time (Root Mean Square Successive Difference; RMSSD) and frequency (low-frequency HRV; LF-HRV (0.04–0.15 Hz), high-frequency HRV; HF-HRV (0.15–0.4 Hz)) domain measures of HRV were then obtained using HRV Analysis Software (Niskanen et al., 2004). According to the Task Force guidelines (1996), RMSSD reflects the integrity of vagus nerve-mediated autonomic control of the heart, HF-HRV reflects parasympathetic activity, and LF-HRV is proportional to sympathetic activity but influenced by parasympathetic tone.

### Procedure

The study was conducted at the Department of Psychology, University of Rome. Participants were asked to refrain from (a) eating, (b) drinking alcohol, tea, or coffee, and (c) strenuous exercise 2 h preceding the scheduled appointment. Participants were seated in a comfortable chair in the experimental room. After providing written informed consent, participants first filled out the series of online questionnaires.

A brief semi-structured interview was conducted in order to assess the nature and the intensity of the meta-emotional problem (e.g., “How childish do you evaluate yourself?”). Specifically, subjects were asked to define what they thought about their phobic reaction, and why it was problematic for them, and to rate how much they believed such evaluation to be true for them on a Likert scale from 0 (completely false for me) to 7 (completely true for me). This interview was used to clinically assess the meta-emotional problem and determine the focus of the Double Standard Technique.

After this interview, subjects were then instrumented for cardiovascular monitoring. Half of the sample (*n* = 16) was randomly assigned to the experimental condition (modulation of the meta-emotional problem), while the other participants (*n* = 17) were assigned to the control condition (i.e., 5 min rest). As shown in **Table 1**, the two groups were matched for age and gender. The Double Standard is a cognitive technique that examines the nature and rationale of applying one standard to the self and a more tolerant standard to others (Leahy, 2003). It can be used to modify dysfunctional thoughts about the Self. It generally consists of asking the patient to express an evaluation on his/her own defect and evaluate how he/she (or people that he/she regards with esteem) would judge another person with the same problem (see **Appendix** for the exact script that has been used in the present experiment). The technique was administered by a licensed cognitive-behavioral psychotherapist.

**Table 1:** Baseline differences between the two experimental groups.

	Double standard (*n* = 16)	Control (*n* = 17)	*t/*χ^2^	*p*
Age	28.4 (10.5)	29.3 (9.4)	0.26	0.79
Gender	14 F, 2 M	13 F, 4 M	0.67	0.41
Phobia	1Be, 1D, 3C, 1I, 1G, 1P, 4S, 4B, 1Sn	3Be, 1D, 1L, 1G, 1P, 4S, 2B, 1Sn	8.64	0.64
Comorbidies	11 No, 5 Yes	10 No, 6 Yes	0.14	0.71
Meta-emotional problem intensity	6 (1.6)	6 (1.7)	0.01	0.99
STAI	45.7 (7.3)	45.1 (9.2)	-0.24	0.81
DS-R	60.1 (4.2)	58.6 (5.1)	-0.95	0.35
Objectively dangerous	2.7 (1.2)	2.3 (0.9)	-1.03	0.31
HR	84.5 (8.6)	85.8 (10.5)	0.39	0.70
RMSSD	37.3 (13.8)	35.2 (12.3)	-0.46	0.65
HF-HRV	48.7 (15.9)	52.2 (19.0)	0.57	0.58
LF-HRV	42.9 (17.8)	40.0 (19.2)	-0.45	0.66

*F, females; M, males; Be, bees; D, dogs; C, cats; I, insects; G, grasshoppers; P, pigeon, S, spiders; 4 B, bugs; Sn, snake.*

For each participant in the experimental group, the target of the Double Standard was the evaluation of their own phobia that emerged during the semi-structured interview (e.g., I evaluate myself as childish). Participants assigned to the control condition were asked to rest quietly with their eyes open for 5 min. The experimental protocol consisted of: (a) 5 min rest, (b) subjective rating of emotions (Visual Analog Scales; VAS), (c) 30-s video, (d) subjective rating of emotions (VAS), (e) 5 min double standard vs. 5 min rest, (f) subjective rating of emotions (VAS), (g) 5 min recovery period, (h) 30-s video, (i) subjective rating of emotions (VAS), (j) 5 min recovery period, (k) subjective rating of emotions (VAS), (l) administration of the Structured Clinical Interview for DSM-IV Axis II Personality Disorders, (SCID-II), (m) debriefing and compensation. The total duration of the experimental protocol was identical for both conditions, i.e., 21 min plus the time needed to complete the VAS and the SCID interview.

### Data Analysis

Data processing was performed using Statistica 8.0 (StatSoft. Inc, USA). To control for the presence of preexisting differences between the two experimental subgroups, two sample *t-*tests were computed on the following variables: age, intensity of meta-emotional problem, evaluation of objective danger, obsessive-compulsive tendencies, trait anxiety, disgust sensitivity, baseline levels of each emotion, HR, RMSSD, HF-HRV, and LF-HRV. Chi square comparison was conducted for gender, type of phobia, and presence or absence of comorbidities.

Low and HF-HRV were natural-log transformed (*ln*) because their distributions were highly skewed. To test for the effectiveness of the phobic induction, a series of *t*-test were computed on HR, RMSSD, and each reported emotion as a function of Time (Before vs. After the first video presentation).

To test for the efficacy of the Double Standard Technique in the experimental group, a paired *t*-test (Before vs. After the Double Standard) was conducted on scores at the first (“How [core evaluation of their meta-emotional problem] do you rate yourself, in a scale of 1–10?”) and the last (“How [core evaluation of their meta-emotional problem] do you rate yourself now, in a scale of 1–10?”) questions of the Double Standard Technique (see **Appendix**).

Two 2 × 2 General Linear Models (GLMs) with Group (Double Standard vs. Control) as a between subject variable and Presentation (First, Second) as a within-subject variable were conducted on HR and RMSSD *during* exposure to the videos. Frequency domain measures were not included as dependent variables in this analysis due to the short duration of video exposure (i.e., 30 s). In fact, the Task Force guidelines report that 1 min is needed to assess the HF components of HRV while approximately 2 min are needed to address the LF component. A second series of 2 × 2 GLMs with Group (Double Standard vs. Control) as a between subject variable and Presentation (First, Second) as a within-subject variable were conducted on HR, RMSSD, HF-HRV, LF-HRV, each considered emotion (VAS), and participants’ evaluation of danger *after* exposure to the videos. To control for individual differences, baseline levels of the examined physiological and emotional variables were included in each model as a covariate.

Eta-squared (η^2^) was calculated to quantify the effect sizes of significant results.

## Results

Initial data screening revealed no significant baseline differences between the two experimental groups (see **Table 1**). Moreover, there were no differences between the two groups regarding the type of evaluation about their phobia (meta-emotional problem): almost the totality of participants in both groups reported their phobia to be problematic because they saw it as a sign of weakness or irrationality.

The first video was effective in inducing a phobic response, as showed by significant changes in HR [*t*(31) = -4.65; *p* < 0.0001], RMSSD [*t*(32) = 3.56; *p* = 0.001] during the videos, and levels of being Happy [*t*(32) = 2.47; *p* = 0.02], Angry [*t*(32) = -2.20; *p* = 0.03], Calm [*t*(31) = 3.96; *p* < 0.0001], Dirty [*t*(32) = -2.71; *p* = 0.01], and Disgusted [*t*(32) = -6.73; *p* < 0.0001], immediately after the exposure to the videos. Changes in levels of being Angry and Happy did not survive Bonferroni correction. Means, standard differences, and statistical results for all the examined variables are shown in **Table 2**

**Table 2:** Effectiveness of the first video in inducing a phobic response on the examined variables.

	*M (SD)_b_*	*M (SD)_v1_*	*t*	*P*
Happy	48.2 (19.9)	37.9 (25.1)	2.47	0.02
Sad	19.4 (21.7)	18.6 (21.6)	0.25	0.80
Anxious	40.8 (27.5)	48.3 (29.9)	-1.38	0.18
Angry	16.5 (23.9)	20.2 (24.9)	-2.20	0.03
Disgusted	11.4 (22.2)	54.0 (31.5)	-6.73	<0.0001
Calm	46.0 (23.3)	32.3 (26.0)	3.96	<0.0001
Dirty	9.5 (17.2)	21.7 (29.8)	-2.71	0.01
Embarassed	28.5 (24.6)	25.2 (25.9)	0.90	0.37
Ashamed	21.1 (22.9)	24.9 (26.6)	-0.90	0.38
HR	85.2 (9.5)	36.2 (12.9)	-4.65	<0.0001
RMSSD	36.2 (12.9)	31.6 (11.4)	3.56	0.001

**M*, mean; *SD*, standard deviation; _b_, during baseline; _v1_, during (HR, RMSSD) or at the end of (VASs) video 1.*

The Double Standard technique resulted effective in reducing the meta-emotional problem [*t*(15) = 4.47; *p* < 0.0001].

**FIGURE 2:**
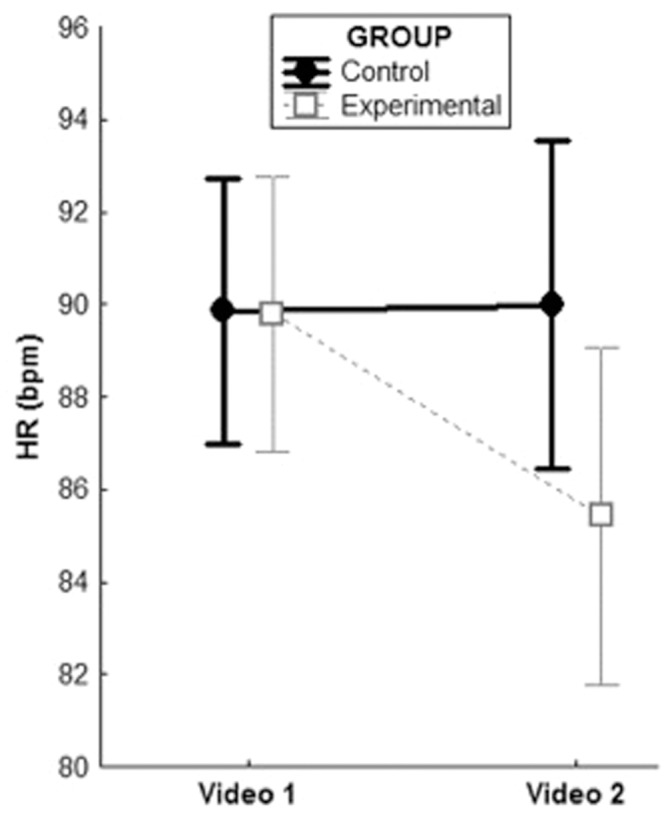
**Group × Presentation interaction for HR during exposures to the videos, controlling for baseline HR**.

As to the GLMs, a significant Group × Presentation interaction emerged for HR during exposure to the videos, *F*(1,29) = 5.52, *p* = 0.03; η^2^= 0.14. *Post hoc* analyses showed no differences between the two groups during the first exposure (*M* = 90.2, *SD* = 9.7 and *M* = 89.2, *SD* = 10.4 for the experimental and control group, respectively), but confirmed diminished HR responses in the experimental group (*M* = 85, *SD* = 12.1) compared to controls (*M* = 90.1, *SD* = 9.3) during the second exposure (*p* = 0.02); see **Figure 2**). The main effects of Group or Presentation were not significant. A marginally significant main effect of Presentation [*F*(1,29) = 3.77, *p* = 0.06] but no significant effects of Group or Group × Presentation emerged for RMSSD.

**FIGURE 3:**
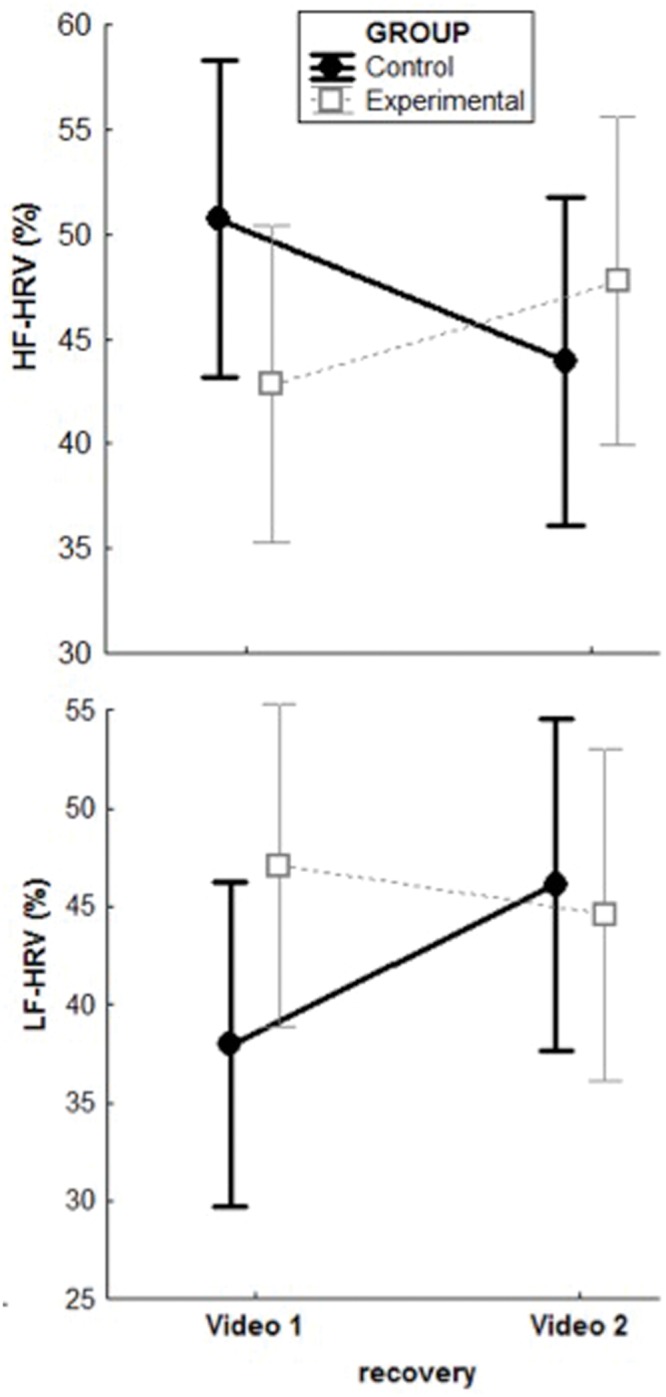
**Group × Presentation interactions for HRV-related parameters after exposures to the videos, controlling for baseline levels**.

Significant Group × Presentation interactions emerged for LF-HRV, *F*(1,29) = 4.74, *p* = 0.04; η^2^= 0.12 and HF-HRV, *F*(1,29) = 13.16, *p* = 0.001; η^2^= 0.28 *after* exposure to the videos. As to LF-HRV, *post hoc* analyses showed a significant increase in controls (*M* = 37.5, *SD* = 12.5 to *M* = 45.6, *SD* = 15.6; *p* = 0.004) but not in the experimental group (*M* = 47.5, *SD* = 19.5 to *M* = 45.5, *SD* = 22.6; *p* = 0.07) after the second compared to the first video exposure. The HF-HRV pattern was the opposite for the experimental and the control groups with a significant increase in the former (*M* = 42.3, *SD* = 14.6 to *M* = 47, *SD* = 17.5; *p* = 0.04) and a significant decrease in the latter (*M* = 51.5, *SD* = 15.6 to *M* = 44.4, *SD* = 15.6; *p* = 0.002). The HF-HRV increase in the experimental group did not survive Bonferroni correction. **Figure 3** depicts the results of the significant interactions for recovery values.

No significant effects of Presentation, Group or Group × Presentation interaction emerged for levels of feeling Sad (*F_s_* < 0.87; *p_s_* > 0.36), Happy (*F_s_* < 0.18; *p_s_* > 0.67), Anxious (*F_s_* < 0.55; *p_s_* > 0.46), Embarrassed (*F_s_* < 2.83; *p_s_* > 0.10), Angry (*F_s_* < 0.43; *p_s_* > 0.52), Calm (*F_s_* < 0.49; *p_s_* > 0.49), Dirty (*F_s_* < 2.62; *p_s_* > 0.12), Disgusted (*F_s_* < 0.39; *p_s_* > 0.54), and Ashamed (*F_s_* < 0.83; *p_s_* > 0.37).

Baseline value was a significant covariate in all the examined models (*ps* < 0.05). Importantly, including the presence of a comorbid depression disorder (yes/no) as an additional covariate did not change our results significantly.

Simple correlation analyses of delta values were performed to test for associations between the amount of reduction in meta-emotional problem and (a) the levels of objectively perceived danger and (b) reduction of physiological responses during exposure. Interestingly, a marginally significant association emerged between changes in the meta-emotional problem and how objectively dangerous participants perceived the object of their phobia (*r* = -0.34; *p* = 0.065). Moreover, changes in the meta-emotional problem were significantly correlated with the amount of changes in LF-HRV (*r* = -0.40; *p* = 0.04) and marginally correlated with changes in HF-HRV (*r* = 0.32; *p* = 0.08). No significant associations emerged for HR.

## Discussion

The goal of the present study was to test whether reducing negative evaluations (secondary or meta-emotional problem) of a specific negative emotional reaction (primary problem) impacted the experience of the emotional reaction itself. Although the effects of meta-cognitions and meta-emotions on primary cognitions and emotions have long been described (Norman and Furnes, 2016 for a review), to the best of our knowledge, no studies directly investigated the relationship between secondary evaluations and emotional reactions in phobic patients.

In this study, reducing the meta-emotional problem had the effect of reducing the physiological but not the subjective symptoms of anxiety during and after phobic exposure. Therefore, results partially supported the hypothesis of a causal role of the meta-emotional problem in phobic reaction. More specifically, results provided preliminary support to the notion that reducing meta-emotional problem contributes to decrease the autonomic arousal (via decreased HR and increased HRV) to the phobic stimulus. Consistent findings have been reported by Rockliff et al. (2008). In their study, low trait self-criticism subjects who received an imagery-based intervention (i.e., the Compassionate Image; Gilbert and Procter, 2006), aimed to reduce self-criticism, showed increased HRV. However, self-criticism can be considered as a broader concept compared to the meta-emotional problem. In fact, self-criticism has been mostly described as a trait, and it has been explored in relation to general life failures and setbacks. Our study has provided preliminary support to the notion that even a basic phobic response can be modulated by changing the appraisal of the emotional reaction from an “abnormal,” “incomprehensible” and “stupid” one, to a more “comprehensible” and “acceptable” one.

Contrary to our expectation, subjective ratings of arousal did not change following the cognitive intervention and remained as high as at baseline in both groups. This result is in contrast to Sartory et al. (1977) who found a strong positive linear relationship between HR changes and subjective fear ratings at intense fear levels but is in agreement with a series of studies that reported a discrepancy between physiological and subjective measures in phobics. For example, animal phobics reported a strong increase in subjective anxiety after forced exposure, without accompanying changes in prolactin levels (Nesse et al., 1980). The same authors replicated this result showing no correlations between HR, blood pressure, plasma levels of adrenaline, noradrenaline, cortisol and growth hormone, and state anxiety with the subjective reports being stronger compared to the physiological ones (Nesse et al., 1985). More recently, Van Duinen et al. (2010) showed a strong dissociation between subjective arousal and cortisol response to spider phobia. The same pattern was found in driving phobia by Alpers et al. (2003). The lack of correspondence between subjective and objective arousal is also supported by correlational research with *r*_s_ < 0.25 in high anxious individuals (e.g., Mauss et al., 2003). The present study does not experimentally identify the causes of such discrepancy. According to our interpretation of the phenomenon, the belief to be a fearful person is extremely credible for the patient, therefore it guides introspection in a confirmatory way, eluding the sensorial information that would justify the change. The double standard technique is likely to reduce the negativity of the evaluation but not its credibility.

Several limitations need to be acknowledged. The major weakness of the study is that our control condition was a passive one (i.e., rest). This constrains the scope of this study as other variables might have explained the reduction in arousal, for example just receiving a form of treatment vs. not receiving any treatment. Despite this shortcoming, the present study provides a set of preliminary results that are promising from a clinical perspective and therefore can lead to a larger research program. Future research should indeed include a third condition in which participants perform a task requiring some reflection about their phobias, without impacting their meta-emotional self-evaluations. Strictly related to this limitation, the present study did not include a measure of the meta-emotional problem before and after the 5-min rest condition in the control group. From a statistical point of view, the adequate way to test for the efficacy of the Double Standard condition would have been to directly compare its effects with those of the 5-min rest condition. We chose not to do so for several reasons: first, it is implausible to think that the meta-emotional problem would have changed (in particular decreased) during a 5-min resting period. More importantly, asking phobic patients twice in a 5-min interval, for example: “Think about your fear of *pigeons*, how *stupid* do you rate yourself, in a scale of 1–10?” would have likely directed their attentional focus on their meta-emotional problem, eliciting ruminative and worrisome thoughts about it, and therefore possibly increasing the anxiety symptomatology (e.g., Ottaviani and Shapiro, 2011). In our opinion, this would have artificially inflated our results. Third, there are ethical reasons why it is important not to direct patients’ attention on their psychological vulnerabilities and then present them again with the object of their phobia.

Second, the sample is quite small, which makes impossible to understand the effects of different rates of self-criticism tendencies and of subpopulations responding differently to the interventions. Moreover, women were twice as much as men in our sample. Although this reflects the fact that specific phobia is more frequent in women compared to men, it may have biased some of the results. Therefore, caution is needed in generalizing results to males.

Third, all participants in our sample were characterized by the presence of the secondary problem. This prevented us to test if the initial response to the phobic stimulus would be different in those who have and those who do not have this additional problem.

Fourth, in light of the dissociation between changes on the physiological and the subjective measures, it would have been greatly informative to assess whether the meta-emotional problem re-occurred after the second exposure, especially given that this exposure elicited very negative subjective emotions. However, the aim of the present study was to test if a reduction of the meta-emotional problem would be effective in reducing the affective and physiological response to the phobic stimulus. Being the reduction of the meta-emotional problem our experimental manipulation and not our dependent variable, unfortunately we did not include a post-assessment of this variable after the second video.

Lastly, only short-term effects were examined, thus limiting the clinical meaning of present findings.

Future studies should overcome these limitations and further explore the role of the meta-emotional problem in different psychopathological disorders in comparison of healthy individuals. It would be interesting to evaluate if and how the secondary or meta-emotional problem contributes to the maintenance of the primary problem and not only to its enhancement. Moreover, the role played by early life experiences of emotional invalidation (Linehan, 1993), such as being ridiculed or debased for the fear of dogs, in the development of the meta-emotional problem should be explored.

As a clinical implication, the presence of the meta-emotional problem should be assessed in order to determine if exposure therapy for specific phobias would benefit from the addition of cognitive intervention. It would be important to clarify if and how the presence of the secondary or meta-emotional problem represents an obstacle to the successful overcoming of the primary problem, in order to establish the therapeutic priorities: the secondary problem leaves room for the resolution of the primary problem or is it necessary to start by shaping it?

## Author Contributions

All authors substantially contributed to the conception and design of the work and to data interpretation. CO, NP, RT, KT ran the experimental sessions, acquired, and analyzed the data. CO, NP, FM, AC drafted the manuscript. CB created the videos.

## Conflict of Interest Statement

The authors declare that the research was conducted in the absence of any commercial or financial relationships that could be construed as a potential conflict of interest.

## Footnotes

1 www.QuestionPro.com

## APPENDIX

### Questions of the Double Standard technique

1. Think about your fear of _____, how ____ do you rate yourself, in a scale of 1 to 10?
2. Now, think about a friend of yours, which is similar to you and that you respect. Please, imagine that also your friend is frightened from ____. How ____ do you rate him/her, in a scale of 1 to 10?
3. Now, think about three persons that know both you and your friend. How ____ would they rate your friend for his/her fear of ____, in a scale of 1 to 10?
4. Now, think about the same three persons. How ____ would they rate you for your fear of ____, in a scale of 1 to 10?
5. Now, think again about your fear of ____, how ____ do you rate yourself now, in a scale of 1 to 10?
